# The fungal symbiont of *Acromyrmex* leaf-cutting ants expresses the full spectrum of genes to degrade cellulose and other plant cell wall polysaccharides

**DOI:** 10.1186/1471-2164-14-928

**Published:** 2013-12-28

**Authors:** Morten N Grell, Tore Linde, Sanne Nygaard, Kåre L Nielsen, Jacobus J Boomsma, Lene Lange

**Affiliations:** 1 Department of Biotechnology, Chemistry and Environmental Engineering, Aalborg University, A.C. Meyers Vænge 15, DK-2450, Copenhagen, Denmark; 2 Department of Biotechnology, Chemistry and Environmental Engineering, Aalborg University, Sohngårdsholmsvej 49, DK-9000, Aalborg, Denmark; 3 Centre for Social Evolution, Department of Biology, University of Copenhagen, Universitetsparken 15, DK-2100, Copenhagen, Denmark

**Keywords:** *Acromyrmex echinatior*, Attine ants, Biomass conversion, Carbohydrate-active enzymes (CAZymes), DeepSAGE, Fungus garden, *Leucocoprinus gongylophorus*, Symbiosis, Transcript profiling

## Abstract

**Background:**

The fungus gardens of leaf-cutting ants are natural biomass conversion systems that turn fresh plant forage into fungal biomass to feed the farming ants. However, the decomposition potential of the symbiont *Leucocoprinus gongylophorus* for processing polysaccharides has remained controversial. We therefore used quantifiable DeepSAGE technology to obtain mRNA expression patterns of genes coding for secreted enzymes from top, middle, and bottom sections of a laboratory fungus-garden of *Acromyrmex echinatior* leaf-cutting ants.

**Results:**

A broad spectrum of biomass-conversion-relevant enzyme genes was found to be expressed *in situ*: cellulases (GH3, GH5, GH6, GH7, AA9 [formerly GH61]), hemicellulases (GH5, GH10, CE1, GH12, GH74), pectinolytic enzymes (CE8, GH28, GH43, PL1, PL3, PL4), glucoamylase (GH15), α-galactosidase (GH27), and various cutinases, esterases, and lipases. In general, expression of these genes reached maximal values in the bottom section of the garden, particularly for an AA9 lytic polysaccharide monooxygenase and for a GH5 (endocellulase), a GH7 (reducing end-acting cellobiohydrolase), and a GH10 (xylanase), all containing a carbohydrate binding module that specifically binds cellulose (CBM1). Although we did not directly quantify enzyme abundance, the profile of expressed cellulase genes indicates that both hydrolytic and oxidative degradation is taking place.

**Conclusions:**

The fungal symbiont of *Acromyrmex* leaf-cutting ants can degrade a large range of plant polymers, but the conversion of cellulose, hemicellulose, and part of the pectin occurs primarily towards the end of the decomposition process, i.e. in the bottom section of the fungus garden. These conversions are likely to provide nutrients for the fungus itself rather than for the ants, whose colony growth and reproductive success are limited by proteins obtained from ingesting fungal gongylidia. These specialized hyphal tips are hardly produced in the bottom section of fungus gardens, consistent with the ants discarding old fungal biomass from this part of the garden. The transcripts that we found suggest that actively growing mycelium in the bottom of gardens helps to maintain an optimal water balance to avoid hyphal disintegration, so the ants can ultimately discard healthy rather than decaying and diseased garden material, and to buffer negative effects of varying availability and quality of substrate across the seasons.

## Background

The Neotropical leaf-cutting ants owe their impressive ecological footprint to an obligate symbiotic relationship with the basidiomycete fungus *Leucocoprinus gongylophorus* that they culture for food in subterranean nest cavities—so-called fungus gardens [1-4]. The ants in return provide the fungus with protection and a continuous supply of freshly-cut leaves as substrate for fungal growth, material that they normally deposit on the uppermost edges of the garden [3,5,6]. To accelerate the subsequent decomposition process, the ants chew the leaf fragments into small pieces and mix the leaf-pulp with fecal droplets [7,8]. This fluid contains substantial quantities of enzymes that the ants ingested with fungal material but without digesting them [7-9], so that new hyphal growth can quickly access the most valuable resources inside the plant cells [10]. Studies by Schiøtt et al. [7] and De Fine Licht et al. [8] have shown that expression of these enzymes tends to be upregulated in the fungal gongylidia, the unique inflated hyphal tips that are harvested by the ants [11,12]. Most notable among these ant-vectored enzymes are proteases, cellulases acting on amorphous cellulose, laccases, and pectinases [7-9,13,14].

**Figure 1:**
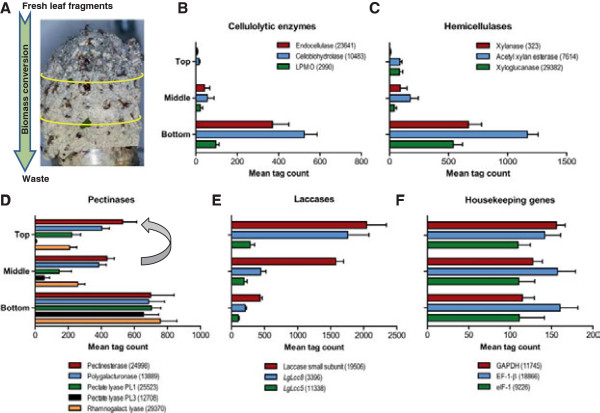
**Fungus garden expression profiles of selected biomass-conversion and housekeeping genes.** Genes were identified and their expression profiles obtained by DeepSAGE analysis on the three sections of the ant fungus garden (mean number of mono-tags + SE). The genes are labeled by their inferred gene function with their mono-tag id in parenthesis. **(A)** A fungus garden of a laboratory colony of the leaf-cutting ant *Acromyrmex echinatior*. The inverted plastic beaker normally covering the garden has been removed before taking the photo. The subdivision of the garden into three sections (top, middle, bottom) is indicated by the yellow concentric rings. Samples for RNA extraction were taken from the center of each section (Photo courtesy of David Nash, University of Copenhagen). **(B)** Expression profiles of three genes encoding cellulolytic enzymes attacking the crystalline cellulose microfibrils. LPMOs (lytic polysaccharide monooxygenases) of family AA9 (formerly family GH61) attack the microfibrils using reactive oxygen species. **(C)** Expression profiles of three genes encoding hemicellulases each containing a CBM1. **(D)** Expression profiles of five genes encoding pectinolytic enzymes. The pectinesterase [GenBank:HQ174766] and the polygalacturonase [GenBank:HQ174767] were previously identified as two of the major fecal fluid pectinases that the ants transfer from the gongylidium-rich middle section to the top section of the garden (curved arrow) [7]. The pectate and rhamnogalacturonan lyases were not among the fecal fluid enzymes and are thus inferred to be active in the section of the garden where they are expressed. **(E)** Expression profiles of three genes encoding laccases. The laccases include *LgLcc8* [GenBank:JQ307230] and *LgLcc5* [GenBank:JQ307227] [8]. The small subunit laccase has not previously been described. **(F)** Expression profiles of three housekeeping genes that were not significantly differentially expressed among the garden sections (see Results). GAPDH, glyceraldehyde 3-phosphate dehydrogenase; EF-1-β, elongation factor 1-β; eIF-1, eukaryotic translation initiation factor 1.

Leaf-cutting ant fungus-farming is reminiscent of a conveyor belt procedure where the ants always add new substrate at the top of the garden, the gongylidia are primarily produced in the middle section, and the ants discard old fungal biomass and substrate residue from the bottom [5,8,10,15]. Under laboratory conditions, it takes ca. six weeks for top section substrate to have reached the bottom from where the ants will move it to a dump outside the nest. Three distinct garden sections can normally be identified: a top section (1), which is grey-green because intact fresh leaf fragments are more abundant than fungal hyphae; a middle section (2), which is white because leaf fragments are no longer visible and fungal growth is abundant; and a bottom section (3), which is grey-brown as only fungal hyphae and plant substrate residue remain (Figure 1A).

There is little doubt that cell proteins and starch are the initial targets when decomposition starts [16,17], but the extent to which the fungal symbiont also decomposes significant quantities of plant cell wall polymers is controversial: Cellulose is both the most abundant and the most challenging of these polymers, but studies disagree on the extent to which cellulose is degraded by the fungus-garden symbiont. Schiøtt et al. [15] have shown that cellulases are primarily present in the top and bottom sections of *Acromyrmex echinatior* fungus gardens and that cellulases are present also in a range of other ant fungus gardens [17]. In contrast, Erthal et al. [18] only detected very low cellulase activity in the top section of *A. subterraneus* fungus gardens and Abril and Bucher [19,20] inferred that cellulose is not converted in fungus gardens at all. In contrast, Suen et al. [21] showed that a significant fraction of crystalline cellulose is converted in gardens of *Atta colombica*, but that the unknown bacterial community of these gardens is instrumental in at least part of this conversion process. However, this latter result was challenged in a recent study by Moller et al. [10], using a carbohydrate polymer profiling technique to study the sequential changes in plant cell wall polysaccharides along the vertical decomposition gradient in *A. echinatior* fungus gardens, which led to the inference that cellulose and some types of xylan are not degraded to any significant degree while xyloglucan and, especially, pectin are. However, the enzyme activity data presented in that study confirmed that cellulases are active in the garden. Microscopic images published by Nagamoto et al. [22] further showed that non-lignified cell walls are absent in the material dumped by the ants, so these findings do not preclude that the fungal symbiont does express enzyme genes for degrading at least some cellulose.

Basidiomycetous fungi have evolved both enzymatic and oxidative strategies for degrading highly recalcitrant crystalline cellulose [23-25]. The typical cellulolytic enzyme repertoire of white-rot wood- and leaf-litter decomposing fungi first includes a number of lytic polysaccharide monooxygenases of the auxiliary activity family 9 (AA9, formerly glycoside hydrolase family 61 (GH61) [26-28]) that randomly cleave cellulose chains at the surface of the microfibrils. This facilitates access by hydrolytic endocellulases (EC 3.2.1.4; e.g. GH5) and at least one reducing end-acting GH7 (EC 3.2.1.176) and one non-reducing-end-acting GH6 (EC 3.2.1.91) cellobiohydrolase (CBHI and II, respectively). As a result, cellobiose or cello-oligosaccharides from the chain-ends are released, so that extracellular or intracellular β-glucosidases (EC 3.2.1.21; GH1 or GH3) can finally cleave the cello-oligomers into glucose monomers [24,29,30]. Many of the cellulases and hemicellulases involved in decomposing lignocellulosic biomass contain a family 1 carbohydrate binding module (CBM1 [26,27]) that attaches the enzymes to the cellulose microfibrils [31,32].

Gene-expression studies of ant fungus garden material have to date remained limited to a few studies on a single fungal gene [15], a subset of fungal genes [7,8], or the entire bacterial microbiome [21]. In the current study, we used the DeepSAGE (Deep Serial Analysis of Gene Expression) technique to target gene expression patterns of the fungal symbiont in samples taken from a laboratory fungus garden of *Acromyrmex echinatior*, after meticulously removing ants, eggs, pupae, and larvae. DeepSAGE is a global, digital transcript-profiling technology, which particularly facilitates the identification of rare transcripts [33] by producing unique 21 bp cDNA tags (mono-tags) from virtually all mRNA molecules in a sample before high-throughput sequencing. Because identical mRNA molecules produce identical tags, the frequencies of these specific sequences are proportional to the expression levels of the corresponding genes. We thus identified the genes that were expressed in the top, middle, and bottom section of the fungus garden, quantified their specific levels of expression, and obtained the differences in expression level between these garden sections. The results obtained allow us to discuss the biomass conversion potential of the fungal symbiont and to interpret this potential in an evolutionary perspective.

## Results

### Generation of DeepSAGE libraries and annotation of mono-tags

After removing low-abundance ones, 29,732 unique mono-tags remained in the dataset across all libraries [see Additional file 1]. For annotation, the 21 bp sequences were first extended by matching to the *L. gongylophorus* EST library [see Additional files 2, 3, 4, 5, 6, 7, 8 and 9] and a low coverage genome sequence [7,8], after which the matching gene fragments (minimum 121 bp—the tag plus 50 bp on either side) were used in BLASTX searches [34] against the GenBank non-redundant protein sequences database. Hits to proteins in the database were obtained for 683 mono-tags [see Additional file 10], among which we selected genes for further analysis based on their predicted involvement in plant polymer degradation and their differential expression among the three garden-sections. Some full-length coding sequences were obtained from the EST library, but all selected genes were subsequently retrieved from the low coverage genome, which also confirmed the selected EST library hits to be of *L. gongylophorus* origin. The full-length sequences of the selected genes were deposited in the European Nucleotide Archive [EMBL:HG764388-HG764410]. To validate the normalization of libraries the mean frequencies of mono-tags representing “housekeeping” genes were compared between the top, middle, and bottom sections of the fungus garden, which showed that there were no significant differences (Welch ANOVAs: elongation factor 1-β [EMBL:HG764411], 142, 157 and 160, *P* = 0.80; eukaryotic translation initiation factor 1 [EMBL:HG764412], 110, 110, and 111, *P* = 1.0; glyceraldehyde 3-phosphate dehydrogenase [GenBank:HQ174770], 156, 128 and 115, *P* = 0.12).

### Cellulase genes are upregulated in the bottom section of the fungus garden

Generally, genes encoding cell wall degrading enzymes and glucoamylase, cutinases, and lipases reached their highest expression level in the bottom section of the fungus garden (Table 1), so we explicitly tested the extent to which cellulose degrading enzyme genes were upregulated compared to the top section (Table 1). This showed that two different GH5 family members, two different reducing-end-acting cellobiohydrolases (CBHI, GH7), one intracellular β-glucosidase (GH3), and a member of family AA9 (formerly GH61, which comprises copper-dependent lytic polysaccharide monooxygenases) were upregulated 1.5-60 fold.

**Table 1:** Expression levels of selected biomass-conversion enzyme-genes in the three sections of the fungus garden

**Tag id**	**Enzyme function deduced from BLAST hits**	**CAZy family**^**a**^	**Top**	**Middle**	**Bottom**	**Test for equal level *P***	**Fold up, bottom relative to top (95% CI)**
			**Mean tag count (95% CI)**				
23641	Endocellulase	GH5, CBM1	6 (0, 14)	41 (0, 123)	370 (150, 591)	0.021	57.9 (25.21, 268.15)
16339	Endocellulase	GH5	96 (13, 178)	28 (0, 60)	140 (56, 224)	0.025	1.5 (0.71, 3.98)
10483	Cellobiohydrolase (CBHI-I)	GH7, CBM1	14 (0, 28)	56 (0, 158)	524 (349, 698)	2.2e-3	36.4 (20.10, 107.96)
24891	Cellobiohydrolase (CBHI-II)	GH7	449 (13, 885)	51 (0, 137)	1211 (911, 1512)	2.2e-4	2.7 (1.53, 8.43)
20092	β-Glucosidase, intracellular	GH3	23 (1, 45)	12 (0, 33)	58 (38, 79)	7.2e-3	2.6 (1.41, 7.54)
2990	Lytic polysaccharide monooxygenase	AA9	0	21 (0, 60)	96 (53, 140)	0	NA
323	Xylanase	GH10, CBM1	7 (0, 21)	92 (0, 274)	669 (360, 977)	8.0e-3	101.3 (35.68, 1089.35)
7614	Acetyl xylan esterase	CE1, CBM1	88 (38, 137)	175 (0, 395)	1168 (917, 1419)	3.0e-4	13.3 (9.12, 22.92)
18781	Xyloglucanase	GH12	16 (1, 31)	95 (70, 119)	141 (0, 287)	4.3e-4	8.8 (2.32, 29.17)
29382	Xyloglucanase	GH74, CBM1	85 (5, 164)	41 (0, 100)	540 (324, 756)	2.0e-3	6.4 (3.48, 18.33)
17822	Pectinesterase	CE8	77 (0, 159)	39 (0, 81)	209 (128, 290)	4.9e-3	2.7 (1.43, 10.17)
24998	Pectinesterase^b^	CE8	529 (295, 763)	435 (297, 572)	698 (304, 1092)	0.25	1.3 (0.75, 2.19)
13889	Polygalacturonase^b^	GH28	403 (273, 533)	386 (244, 527)	686 (418, 954)	0.075	1.7 (1.16, 2.43)
16820	Pectate lyase^b^	PL1	34 (11, 56)	19 (0, 57)	189 (10, 368)	0.11	5.6 (1.88, 12.58)
25523	Pectate lyase	PL1	222 (74, 370)	146 (0, 381)	704 (547, 860)	8.5e-4	3.2 (2.06, 6.01)
12708	Pectate lyase	PL3	6 (0, 17)	54 (0, 160)	655 (398, 912)	4.1e-3	116.9 (45.09, 1220.07)
29370	Rhamnogalacturonan lyase	PL4	208 (87, 329)	259 (125, 392)	757 (477, 1036)	6.0e-3	3.6 (2.28, 6.45)
17541	Endo-1,5-α-L-arabinanase	GH43	141 (22, 260)	81 (31, 131)	204 (110, 297)	0.043	1.4 (0.79, 3.63)
17220	Glucoamylase	GH15, CBM20	32 (1, 62)	35 (0, 107)	130 (113, 146)	8.6e-4	4.1 (2.40, 11.80)
26445	α-Galactosidase	GH27	162 (79, 245)	163 (93, 233)	660 (578, 742)	5.7e-6	4.1 (2.96, 6.40)
10080	Cutinase	-	433 (0, 889)	367 (156, 577)	960 (743, 1177)	2.4e-3	2.2 (1.24, 7.86)
28797	Cutinase	-	211 (0, 444)	242 (0, 555)	468 (340, 596)	0.067	2.0 (1.18, 9.25)
14730	Esterase/lipase	-	2 (0, 7)	31 (0, 88)	357 (298, 416)	9.9e-5	148.6 (65.90, 1290.24)
1743	Lipase	-	144 (28, 259)	595 (424, 765)	718 (423, 1013)	6.7e-4	5.0 (2.78, 12.01)

^a^Carbohydrate-Active Enzymes family [27]: GH, glycoside hydrolase; AA, auxiliary activity; CBM, carbohydrate-binding module; CE, carbohydrate esterase; PL, polysaccharide lyase.

^b^Identified by Schiøtt et al. [7] [GenBank:HQ174765-HQ174767].

DeepSAGE analysis did not give direct evidence for the presence of non-reducing-end-acting cellobiohydrolases (CBHII, GH6) in any of the garden sections. However, to establish whether the fungus did have a functional GH6 gene, we screened the EST library for GH6 homologs and identified one complete transcript that was 72% identical at the amino acid level to *Agaricus bisporus* cel3AC [GenBank:AAA50608] and predicted to include a CBM1 and thus to specifically bind to cellulose microfibrils [27,31]. Using the peptide pattern recognition (PPR) program [35], the two GH5s could be assigned to EC 3.2.1.4 (endocellulases), but only one of these was predicted to contain a CBM1. The tag count for this gene (id 23641) was low in the top section and more than 50 times higher in the bottom section (Figure 1B), whereas the tag count for the other GH5 gene (id 16339) was not significantly different between top and bottom sections, but significantly lower in the middle section (Table 1). The same pattern was observed for the two GH7s: Expression of the gene containing a CBM1 (id 10483) was more than 30 times higher in the bottom section than in the top of the garden (Figure 1B), while the gene with no CBM1 (id 24891) was only moderately upregulated in the bottom section compared to a high level in the top, but markedly upregulated compared to expression in the middle section (Table 1). Also the tag count for the GH3 gene (id 20092) was significantly higher in the bottom section although expression was much lower than for the GH5s and the GH7s. The mono-tag representing the AA9 (formerly GH61) gene (id 2990) was not detected in the top section, but encountered almost a 100 times in the bottom section (Figure 1B).

### Transcript levels of non-cellulolytic biomass conversion genes

In addition to genes encoding cellulose-active enzymes, a number of other polysaccharide-active enzyme genes were upregulated in the bottom section compared to the top and/or middle sections. These included hemicellulase genes, such as a xylanase (GH10), an acetyl xylan esterase (CE1), two xyloglucanases (GH12, GH74) (Table 1; Figure 1C), and an endo-1,4-β-mannanase (id 12084, GH5) [see Additional file 10], and genes encoding pectinolytic enzymes, such as a pectinesterase (CE8; de-esterifies homogalacturonan), two pectate lyases (PL1, PL3; degrades de-esterified homogalacturonan), a rhamnogalacturonan lyase (PL4; degrades rhamnogalacturonan I backbone), and an endo-1,5-α-L-arabinanase (GH43; degrades rhamnogalacturonan I side chains) (Table 1; Figure 1D). The assignment of GH family members to function was confirmed by PPR [35].

All hemicellulases except the GH12 xyloglucanase (id 18781) were predicted to contain a CBM1 and thus to be anchored to the cellulose microfibrils while performing their activity (as for the CBM1 containing cellulases). The identified GH10 xylanase was different from the GH11 xylanase Lg*Xyn1* identified by Schiøtt et al. [15] for another fungus garden from the same population of *A. echinatior*. Genes encoding a lipase and esterases (cutinases) were also upregulated in the bottom section compared to the top, although lipase expression in the middle section and cutinase expression in both the top and middle sections was also substantial (Table 1). Finally, we detected increased expression of a glucoamylase gene (GH15) and an α-galactosidase gene (GH27) in the bottom section (Table 1), which are known to be involved in the mobilization of starch and the degradation of galactomannans (with endo-1,4-β-mannanase) and other galactosides, respectively.

In addition to genes predicted to be involved in biomass conversion, we also established a list of the most highly expressed genes overall (peak > 1000 tag counts) (Table 2). At the top of this list appeared a gene encoding a cerato-platanin-related secreted protein (id 14513) that reached its maximal expression in the middle section. Cerato-platanin proteins self-assemble into a surface coating layer that enables hyphae to grow into the air and adhere to surfaces [36]. Also a gene encoding a hydrophobic surface binding protein (id 28405) was among the most highly expressed genes across the three garden Sections. A NADH-quinone oxidoreductase gene (id 21633) peaked in the top and middle sections, but was also highly expressed in the bottom section. These genes catalyze the reduction of quinones to hydroquinones and may be involved in Fenton chemistry-mediated degradation of cellulose [23]. A phosphate transporter gene (id 20658) was highly expressed throughout the garden and doubled its expression level in the middle and bottom sections relative to the top. The two most highly expressed carbohydrate degrading enzyme genes, the non-CBM1 GH7 (CBHI-II, id 24891) and acetyl xylan esterase (id 7614), both peaked in the bottom section of the fungus garden and were among the genes with the highest expression in that section. The most prominent secreted enzyme genes that peaked in the top section were two laccases (multicopper oxidases) (Figure 1E), confirming a recent study showing that laccase activity is of crucial importance for phenol detoxification in the top section of the *A. echinatior* fungus gardens [8]. Additional highly expressed genes that were retrieved encoded ubiquitous cytoplasmic proteins such as cyclophilin, ubiquitin conjugating enzyme, and glutathione S-transferase.

**Table 2:** The most highly expressed genes in the fungus garden

**Tag id**	**Enzyme function deduced from blast hits as in Table**1	**Top**	**Middle**	**Bottom**	**Test for equal level *P***
		**Mean tag count (95% CI)**			
14513	Cerato-platanin-related secreted protein	4918 (3465, 6370)	7389 (0, 15778)	4459 (3571, 5347)	0.51
6186	Cyclophilin (peptidyl-prolyl cis-trans isomerase)	2818 (2029, 3607)	3764 (2535, 4993)	2628 (2333, 2922)	0.10
22660	Conserved hypothetical protein	3196 (1902, 4489)	1554 (401, 2707)	1427 (811, 2043)	0.042
21633	NADH-quinone oxidoreductase	2680 (2014, 3346)	2851 (1744, 3958)	1477 (1122, 1831)	7.1e-3
28405	Hydrophobic surface binding protein	2445 (957, 3933)	1108 (715, 1501)	1893 (1488, 2298)	0.011
20658	Phosphate transporter	917 (377, 1457)	2124 (891, 3356)	2293 (1253, 3333)	0.027
19506	Laccase small subunit	2044 (1208, 2881)	1578 (1202, 1955)	433 (336, 529)	4.9e-4
3396	Laccase^a^	1757 (860, 2655)	507 (320, 693)	210 (161, 258)	4.0e-3
3360	Ubiquitin conjugating enzyme	1709 (1389, 2030)	1583 (1161, 2005)	1393 (752, 2035)	0.51
24891	Cellobiohydrolase (CBHI-II) [GH7]	449 (13, 885)	51 (0, 137)	1211 (911, 1512)	2.2e-4
7614	Acetyl xylan esterase [CE1]	88 (38, 137)	175 (0, 395)	1168 (917, 1419)	3.0e-4
19652	Glutathione S-transferase	1147 (701, 1592)	998 (726, 1271)	889 (633, 1146)	0.43

The table includes genes peaking at more than 1000 tag counts, but excludes ribosomal RNA and ribosomal protein genes.

^a^*LgLcc8* identified by De Fine Licht et al. [8] [GenBank:JQ307230].

## Discussion

There has been considerable controversy about the extent to which the symbiosis between leaf-cutting ants and their *L. gongylophorus* symbiont utilizes the recalcitrant polymers of plant cell walls as a source of nutrients [10,15-22]. Our present results show that the fungal symbiont of *A. echinatior* leaf-cutting ants produces a range of green-biomass conversion enzymes. Similar, but not identical, results were obtained in a parallel, recently published study by Aylward et al. [37]. The two studies complement each other, as Aylward et al. used genomics and metaproteomics tools to investigate fungus gardens whereas we used transcript profiling. However, we also present specific new evidence that the fungal symbiont produces a number of transcripts that encode enzymes for degrading crystalline cellulose. This should in principle enable the fungus to fully deconstruct plant cell walls consisting of crystalline cellulose microfibrils, hemicellulose, and pectins and to use mostly the released glucose and xylose as a source of energy. Yet, our transcript profiling data show that these cellulolytic abilities were expressed primarily towards the end of the decomposition process, at a stage where the ants are known to discard old garden material containing substantial amounts of cellulose, as well as significant amounts of older fungal hyphae.

### The fungal symbiont can degrade all plant cell wall and cuticle polymers

The cellulase genes showing the most marked upregulation in the bottom section were the CBM1 encoding genes and the lytic polysaccharide monooxygenase of family AA9 (formerly GH61) (Figure 1B). The highly conserved CBM1 module is commonly found in fungal cellulases, attacking the crystalline microfibrils [26,31]. Family AA9 enzymes have recently been shown to act directly on crystalline cellulose, partially degrading and loosening the structure of the microfibrils while increasing substrate accessibility for the other types of cellulases [25], although some AA9s may be active on other carbohydrates than cellulose [38]. The high expression of a fungal NADH-quinone oxidoreductase suggests that oxidative biomass conversion processes in the garden may not only rely on enzymatic catalysis but also on Fenton reactions, as implicated for other basidiomycetes [23,39]. The high expression level of a gene coding for a secreted cerato-platanin-related protein—the highest expression level of a protein-encoding gene found in our entire study—combined with the high expression of a hydrophobic surface binding protein gene (Table 2) suggests that producing molecules that enable hyphae to grow in the air without losing water is important in all sections of the garden.

We did not directly quantify enzyme abundance and the ants are known to transfer some cellulases from the middle to the top section of gardens via their fecal droplets [9]. This may explain higher activity levels in samples taken from the top of the garden relative to the middle section [15], but does not affect that our results consistently indicate that crystalline cellulose is increasingly exposed to enzyme break down towards the bottom section where the low glucose concentration may act to induce cellulase activity [15]. This conclusion is supported by proteomics data from the Aylward et al. study [37], showing high GH6 and GH7 enzyme production in the bottom section of an *A. echinatior* fungus garden. Particularly the non-CBM1 cellulases showed a dual expression profile, with a peak in the top section where the ants chew fresh leaves into pulp and a more pronounced peak in the bottom section (Table 1). We suspect, however, that these enzymes have different roles when targeting fresh leaf pulp in the top of gardens and residues in the bottom because microscopic imaging has shown that complete degradation of all non-lignified cell walls is achieved only in the refuse dump after the ants have discarded bottom material from their gardens [22].

Similar to the cellulolytic genes, also the expression of genes that encode enzymes for degrading the major leaf hemicelluloses, xylan and xyloglucan, increased substantially in the bottom section of the garden (Figure 1C). This is consistent with the proteomics data of Aylward et al. [37], which indicates a similar regulatory profile of predicted leaf hemicellulases although this is not directly evident from their presented bar charts. However, we only identified few β-xylosidase transcripts [see Additional file 10] indicating that the xylose oligomers produced by xylanase activity are not metabolized to xylobiose or xylose monomers to any significant degree. Xylan therefore seems to be of marginal nutritional value to the fungal symbiont, confirming the conclusion by Moller et al. [10] that cell wall hemicellulose is only partially degraded to facilitate hyphal access to intracellular proteins and starch grains. The fact that *L. gongylophorus* grows very well in pure culture with xylan as the only carbon source, at a rate similar to growing on starch [16], indicates that ample starch must be available in the fresh leaf cells to make decomposition proceed at high speed. Starch may be preferentially targeted relative to xylan because glucose is the preferred carbon source of the ants [40], but the fresh leaves may also provide the symbiosis with so much starch that there is no need to break-down xylan because overall symbiotic performance is ultimately protein-limited rather than sugar-limited (see next section).

Also pectinolytic activity in the fungus garden was bimodally distributed across the three garden sections. Previous results of Moller et al. [10] on fungus gardens of the same species showed that a substantial part of the pectin is degraded in the top section immediately after fresh leaf pulp is deposited, but our present results show that there is even more pectinolytic transcription in the bottom section (Figure 1D). Also this result is consistent with Aylward et al. [37], showing the same general regulatory trend in the products of pectinolytic genes, although this is not clearly stated. Also for these enzymes upregulation in the gongylidia and vectoring via ant fecal droplets may change the final distribution between the middle section (where most gongylidia are) and the top section (where gongylidia-upregulated enzymes are most needed [7,8]) (Figure 1D), but this does not affect the high expression of pectinolytic genes in the bottom section of fungus gardens. This can only be explained by many pectins remaining to be degraded, and that process having been postponed until a relatively late phase. It is consistent with a number of other studies that have suggested that pectin, like hemicellulose, is primarily degraded not as a source of nutrients, but merely to gain access to the intracellular nutrient stores as soon as possible after leaf pulp is deposited at the top of gardens, possibly also by unmasking hemicellulose for enzymatic attack [7,10,16,17,41]. The later peak activity in the bottom of gardens suggests that this decomposition activity is reinstated, but our general knowledge of the biology of this symbiosis makes it unlikely that these two activities serve the same purpose.

Finally, we find a similar pattern for cutinase activity [18], with transcripts being present throughout the garden, but this time with only a small but significant increase in the bottom compared to the top and middle sections (Table 1). This indicates that leaf cuticular material (epidermis cells) in the chewed-up leaf fragments is also targeted by the fungus, and increasingly so towards the bottom section of the garden. However, these cells seem to be a lower decomposition priority, consistent with microscopic observations by Nagamoto et al. [22] that epidermis cells are only partially degraded in the material that the ants discard from the bottom section of their gardens.

### The symbiosis is nitrogen rather than carbon limited

The glucose level in the bottom section of the fungus garden is low [15], presumably because all the easily accessible nutrients have been utilized by the fungus. Still, we find a high level of fungal phosphate transporter transcripts in the bottom section—similar to the level in the highly active middle section—indicating high metabolic activity. We hypothesize that the increased level of cell wall degrading enzymes in the bottom section enables the fungus to access the interior of cells that were not opened earlier in the decomposition process, but that nutrients obtained in this phase serve fungal maintenance and perhaps some final growth. This idea is supported by the observed upregulation of genes encoding enzymes such as lipase (Table 1) and glucoamylase (Table 1) [37] in the bottom section of the garden, indicating that the fungus obtains access to additional intracellular nutrients. At this stage, the fungal symbiont may also utilize cell wall polymers, but this is unlikely to benefit the nutritional symbiosis as the ants are known to discard old garden material from the bottom section containing substantial residues of cellulose and hemicellulose [10]. This interpretation is consistent with the fungal symbiont being able to grow on artificial cellulose media [42] and with our identification of some expression of an intracellular β-glucosidase, suggesting the use of some cellobiose as energy source at this late decomposition stage.

In a previous study on laboratory fungus gardens of the same ant, *A. echinatior*, clear evidence was found that the fungal gongylidia that feed the ants are not present towards the bottom of gardens [8]. This implies that the ants are unlikely to retrieve significant nutritional resources from the bottom of their gardens, consistent with them discarding this material, containing both cellulose and intact fungal hyphae. As the rapidly growing larvae of leafcutter ants only consume gongylidia [12] and *Atta* gardens harbor nitrogen-fixing bacteria [43] it seems likely that the attine fungus-farming symbiosis is nitrogen-limited rather than carbon (glucose)-limited. Any glucose released in the bottom section of a garden may thus primarily serve the need of the fungal symbiont when care by the farming ants is about to be terminated, provided sufficient fresh leaf substrate is brought in at the top of the garden. The fungal symbiont may have retained enzymes for cell wall degradation from its free-living saprophytic ancestors because the optimal time for discarding old mycelium by the ants may depend on the availability and quality of fresh substrate. Bottom-garden-fungus may thus be discarded later in the dry season when plant parts have low starch content [6,14] than in the wet season, an idea that should be easily testable.

To understand how natural selection has shaped enzymatic functions in *L. gongylophorus*, it is important to realize that the fungus has no fitness interests that are independent of the ants, as it completely relies on vertical transmission across generations by winged virgin queens when they leave for their mating flight. This implies that the fungal symbiont has only been under selection to maintain its old mycelium in the bottom of gardens when that benefits the farming ants. We believe that these benefits have too readily been assumed to be generally related to glucose production for the ants, as the putative substrate buffering function may only be important in stressful periods. However, if the symbiosis is not glucose-limited most of the time, and glucose produced in the bottom of gardens cannot be offered to the ants via gongylidia, it would seem more logical to look for indirect benefits. The abundant transcripts that may mediate the maintenance of an optimal water balance in old mycelium (see previous section) suggest that a more generally important buffering mechanism may be at work. Maintaining active growth almost certainly reduces disease pressure in the bottom of fungus gardens, and when that benefit can be achieved without using limiting resources glucose production may represent the ultimate terminal service of the fungus to symbiotic health before it is discarded.

Finally, it is important to realize that some bacterial garden symbionts may also contribute to the conversion processes, whereas others may provide antimicrobial products for keeping the garden free of antagonistic microbes [14,21,44]. A comprehensive understanding of how the fungal and the bacterial roles are combined is still in its infancy, and new data may thus continue to change our understanding of functional complementarity in fungus garden substrate conversion.

## Conclusions

After analyzing genes expressed specifically by the fungus-garden symbiont *in situ*, we conclude that *L. gongylophorus* is producing all enzymes necessary for degrading the major plant-cell-wall polysaccharides: cellulose, hemicellulose, and pectin. By further comparing the expression level of these carbohydrate-active enzyme genes in the top, middle, and bottom sections of the garden, representing consecutive stages in the decomposition of biomass, we show that—except for part of the pectin that is already converted in the top section—the degradation of the cell wall polysaccharides occurs primarily towards the end of the decomposition process. The monosaccharides (mainly glucose) released in these processes, either from hitherto unexploited intracellular nutrient stores or from the polymers themselves, appear to mostly serve the needs of the fungus itself because the nutrient-rich fungal gongylidia that the ants ingest and feed to their rapidly growing larvae are mainly present in the middle section of the garden. The low likelihood of excess glucose being transferred from the bottom section of the garden to the ants is consistent with the symbiosis being limited by the availability of protein-nitrogen in the gongylidia rather than by glucose-carbon. The results of our DeepSAGE analyses of gene expression suggest that the fungal symbiont has retained the ability to degrade recalcitrant plant cell wall polysaccharides in order to maintain active growth even when no longer producing ant food. This may be of use when leaves with higher starch content are unavailable during the dry season, but will also help protect old fungus from disease until it is discarded by the ants. Both possible functions would have stabilized the symbiosis over evolutionary time.

## Methods

### Biological material

Fungus garden samples were taken in May 2009 from *A. echinatior* colony Ae349, which was collected in 2007 in Gamboa, Panama, and established in a climate room at the University of Copenhagen under standard conditions of about 25°C and 70% relative humidity [45]. Ants were supplied with a diet of bramble (*Rubus* spp.) leaves, rice, and pieces of fruit (mainly apple)—switching to a diet of bramble leaves and fruit only in the 6 weeks preceding sampling. Pure cultures of the major fungus garden symbiont, the basidiomycete fungus *Leucocoprinus gongylophorus*, were obtained by inoculating pieces of the fungus garden onto (a) potato dextrose agar (PDA) and (b) 5% w/v wheat bran agar (WBA) plates, both including 200 μg/ml ampicillin. The WBA plates were covered with a thin section of water agar before inoculation. Both types of plates were incubated at 25°C without light. Mycelium was passed onto fresh plates every two weeks. After two months, only *L. gongylophorus* mycelium was present on the plates, as determined by the presence of gongylidia—a unique species identification character of this fungus [11].

### DeepSAGE

To compare expression profiles along the vertical decomposition gradient of the fungus garden, we divided the garden into three sections: top, middle, and bottom (Figure 1A). Total RNA was extracted from five samples of the top section, four samples of the middle section, and five samples of the bottom section (biological replicates). Before RNA extraction, samples were carefully examined under a stereo microscope for the presence of ant eggs, larvae, and pupae, which were all quickly removed using a pair of forceps. Additionally, RNA was extracted from three samples of the PDA pure cultures and five samples of the WBA pure cultures. Fungus garden or pure culture material (100–200 mg) was grinded in liquid nitrogen and total RNA extracted using the RNeasy Plant Mini Kit (QIAGEN). The initial lysing step using either buffer RLT (QIAGEN) or Fenozol (A & A Biotechnology) was followed by two extractions each with one volume of phenol:chloroform:isoamyl alcohol (25:24:1) and then one extraction with one volume of chloroform:isoamyl alcohol (24:1). Finally, the water phase was applied to a QIAshredder spin column and the rest of the steps performed as explained in the RNeasy protocol.

Two μg total RNA from each of the fungus garden and pure culture samples was used to construct section-specific and culture-specific DeepSAGE tag libraries, respectively, as previously described [33,46], but with the following modifications: the ditag formation steps were omitted, and adaptors were added directly to mono-tags for amplification and sequencing. Sequencing was performed with the Cluster Generation Kit (Illumina) and the 36-cycle SBS Reagent Kit (Illumina), using an Illumina Genome Analyzer following manufacturer instructions. Mono-tag sequences were sorted according to the original RNA sample by a unique 3-bp key in the downstream adaptor and the gene-specific part (17 bp unique sequence plus the 4 bp anchoring enzyme recognition site) extracted as previously described [47]. Tags represented 10 times or less were initially removed from the dataset and the remaining tag counts in each library normalized to counts per million to allow comparisons of the relative expression levels among the garden sections. To exclude mono-tags that may have been generated by sequencing errors, only those represented in at least three different libraries and observed 22 times or more across all libraries were analyzed further.

### Preparation of EST library

For preparing a general EST library, 2 μg total RNA from each of the three sections of the garden (top, middle, and bottom) and from the PDA and WBA pure cultures was mixed. Approximately 36 bp of double-stranded cDNA fragments was prepared for sequencing, using the mRNA-Seq Sample Prep Kit (Illumina) according to the manufacturer’s protocol. The cDNA fragments were sequenced as described above for the mono-tags. Contigs were assembled from the 36 bp sequences using Velvet [48] with the following overlaps: 17, 19, 21, 23, 25, 27, 29, and 31 bp, producing 8 different versions of the EST library [see Additional files 2, 3, 4, 5, 6, 7, 8 and 9].

### Statistics

As several measurements were taken from each section, statistical techniques could be used to assess measurement error and evaluate whether differences between sections were larger than could be expected by chance. For each section-tag combination 95% confidence intervals were computed, and the hypothesis of equal expression levels in all three sections was tested by Welch ANOVA, which does not require equal variance across the sections. When all counts in one section were 0, Kruskal-Wallis rank sum test was used instead. Finally, gene expression levels in the bottom section relative to the expression levels in the top section (fold increase bottom relative to top) were computed, using simulation for the corresponding confidence intervals [49].

### PPR methods

Peptide pattern recognition, PPR [35], is a new alignment-independent method for predicting the function of biological sequences by finding functionally and structurally conserved short sequence motifs (n-mers). If the input is too divergent to have a common set of n-mers, PPR will separate the sequences into groups, defined by common n-mers. These features make PPR suitable for comparing large numbers of divergent sequences that are difficult to subgroup with other methods. For predicting the most probable function of the identified GH family members, the PPR program was fed with sequences from the Carbohydrate-Active Enzymes database [26,27], producing subgroups each characterized by the EC number of the subgroup members [35]. The identified GHs from the current study were treated with PPR (n-mer = 5) and the resulting peptides mapped to GH family subgroups. Prediction of function was then based on the subgroup(s) to which the majority of the n-5 peptides belonged.

### Availability of supporting data

The data sets supporting the results of this article are available in the European Nucleotide Archive (ENA) [http://www.ebi.ac.uk/ena/data/view/PRJEB4675] and in this article and its additional files.

## Abbreviations

AA: Auxiliary activity; ANOVA: Analysis of variance; CBH: Cellobiohydrolase; CBM: Carbohydrate-binding module; CE: Carbohydrate esterase; DeepSAGE: Deep serial analysis of gene expression; EST: Expressed sequence tag; GH: Glycoside hydrolase; PDA: Potato dextrose agar; PL: Polysaccharide lyase; PPR: Peptide pattern recognition; WBA: Wheat bran agar.

## Competing interests

The authors declare that they have no competing interests.

## Authors’ contributions

JJB and LL conceived of the study. LL, MNG, and KLN designed and supervised the experimental work. TL carried out the experimental work. SN and TL annotated the mono-tags. MNG assembled all selected genes to full-length, manually curated computer-generated annotations, produced the figure and tables, and wrote the manuscript with input from JJB and LL. All authors read and approved the final manuscript.

## Supplementary Material

Additional file 1 **List of 29732 mono-tag sequences and their normalized tag counts.** The first column is the tag id, followed by the 5′ to 3′ sequence of each mono-tag (except the 4 bp anchoring enzyme recognition site, 5′-CATG that is common to all mono-tags). The following 22 columns display the normalized tag counts in each of the 22 DeepSAGE libraries produced: three PDA *L. gongylophorus* pure culture libraries (1–1 to 1–3), five WBA *L. gongylophorus* pure culture libraries (2–1 to 2–5), five top section libraries (3–1 to 3–5), four middle section libraries (4–1 to 4–4), and five bottom section libraries (5–1 to 5–5). The Top, Middle, and Bottom columns display the mean tag counts for each of the three fungus garden sections. Click here for file

Additional file 2 **Expressed sequence tag library ‘contigs17’.** The 36 bp Illumina reads were assembled using Velvet [48] with 17 bp overlaps. The definition line for each sequence includes the coverage (cov). Click here for file

Additional file 3 **Expressed sequence tag library ‘contigs19’.** The 36 bp Illumina reads were assembled using Velvet [48] with 19 bp overlaps. The definition line for each sequence includes the coverage (cov). Click here for file

Additional file 4 **Expressed sequence tag library ‘contigs21’.** The 36 bp Illumina reads were assembled using Velvet [48] with 21 bp overlaps. The definition line for each sequence includes the coverage (cov). Click here for file

Additional file 5 **Expressed sequence tag library ‘contigs23’.** The 36 bp Illumina reads were assembled using Velvet [48] with 23 bp overlaps. The definition line for each sequence includes the coverage (cov). Click here for file

Additional file 6 **Expressed sequence tag library ‘contigs25’.** The 36 bp Illumina reads were assembled using Velvet [48] with 25 bp overlaps. The definition line for each sequence includes the coverage (cov). Click here for file

Additional file 7 **Expressed sequence tag library ‘contigs27’.** The 36 bp Illumina reads were assembled using Velvet [48] with 27 bp overlaps. The definition line for each sequence includes the coverage (cov). Click here for file

Additional file 8 **Expressed sequence tag library ‘contigs29’.** The 36 bp Illumina reads were assembled using Velvet [48] with 29 bp overlaps. The definition line for each sequence includes the coverage (cov). Click here for file

Additional file 9 **Expressed sequence tag library ‘contigs31’.** The 36 bp Illumina reads were assembled using Velvet [48] with 31 bp overlaps. The definition line for each sequence includes the coverage (cov). Click here for file

Additional file 10 **List of 683 mono-tag sequences for which we obtained one or more hits to proteins in the databases.** The first column is the tag id, followed by the 5′ to 3′ sequence of each mono-tag (except the 4 bp anchoring enzyme recognition site, 5′-CATG that is common to all mono-tags). The following 22 columns display the normalized tag counts in each of the 22 DeepSAGE libraries produced: three PDA *L. gongylophorus* pure culture libraries (1–1 to 1–3), five WBA *L. gongylophorus* pure culture libraries (2–1 to 2–5), five top section libraries (3–1 to 3–5), four middle section libraries (4–1 to 4–4), and five bottom section libraries (5–1 to 5–5). The Top, Middle, and Bottom columns display the mean tag counts for each of the three fungus garden sections. The final column shows the inferred function based on the most significant BLASTX hit(s) obtained for each mono-tag after first extending the sequence by searching our EST library and a *L. gongylophorus* low coverage genome sequence [7,8]. Click here for file
